# MicroRNAs and Long Non-coding RNAs in c-Met-Regulated Cancers

**DOI:** 10.3389/fcell.2020.00145

**Published:** 2020-03-11

**Authors:** Hong Zhan, Sheng Tu, Feng Zhang, Anwen Shao, Jun Lin

**Affiliations:** ^1^Women's Hospital, School of Medicine, Zhejiang University, Hangzhou, China; ^2^State Key Laboratory for Diagnosis and Treatment of Infectious Diseases, Collaborative Innovation Center for Diagnosis and Treatment of Infectious Diseases, The First Affiliated Hospital, Zhejiang University School of Medicine, Hangzhou, China; ^3^School of Medicine, Zhejiang University Hangzhou, Hangzhou, China; ^4^Department of Neurosurgery, The Second Affiliated Hospital, Zhejiang University School of Medicine, Hangzhou, China

**Keywords:** microRNAs, long non-coding RNAs, hepatocyte growth factor, c-Met, cancer, drug-resistance

## Abstract

MicroRNAs (miRNAs) and long non-coding RNAs (lncRNAs) are components of many signaling pathways associated with tumor aggressiveness and cancer metastasis. Some lncRNAs are classified as competitive endogenous RNAs (ceRNAs) that bind to specific miRNAs to prevent interaction with target mRNAs. Studies have shown that the hepatocyte growth factor/mesenchymal-epithelial transition factor (HGF/c-Met) pathway is involved in physiological and pathological processes such as cell growth, angiogenesis, and embryogenesis. Overexpression of c-Met can lead to sustained activation of downstream signals, resulting in carcinogenesis, metastasis, and resistance to targeted therapies. In this review, we evaluated the effects of anti-oncogenic and oncogenic non-coding RNAs (ncRNAs) on c-Met, and the interactions among lncRNAs, miRNAs, and c-Met in cancer using clinical and tissue chromatin immunoprecipition (ChIP) analysis data. We summarized current knowledge of the mechanisms and effects of the lncRNAs/miR-34a/c-Met axis in various tumor types, and evaluated the potential therapeutic value of lncRNAs and/or miRNAs targeted to c-Met on drug-resistance. Furthermore, we discussed the functions of lncRNAs and miRNAs in c-Met-related carcinogenesis and potential therapeutic strategies.

## Introduction

**Figure 1:**
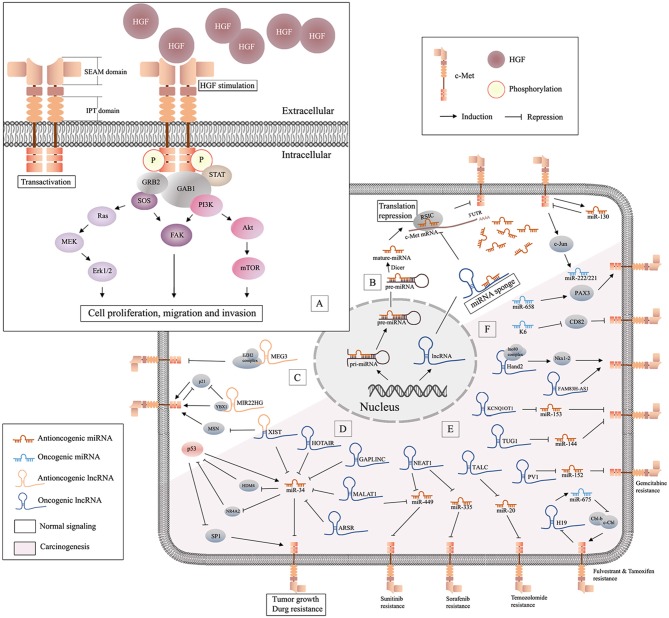
Schematic of HGF/c-Met signaling and lncRNA/miRNA/c-Met interaction. **(A)** Binding of HGF initiates stable c-Met homodimerization, which activates various downstream signaling pathways; **(B)** miRNAs are generated from the nucleus and can be decoyed by specific lncRNAs in cytoplasm, and antioncogenic miRNAs may suppress tumor progression by repressing the translation of c-Met mRNA; **(C)**, illustration of antioncogenic lncRNAs (MEG3 and MIR22HG) in the regulation of c-Met; **(D)** the graphical representation of p53/miR-34 and lncRNA/miR-34/c-Met pathway; **(E)** oncogenic lncRNAs/miRNAs/c-Met in drug resistance; **(F)** oncogenic miRNAs in c-Met related carcinogenesis.

Mesenchymal-epithelial transition factor (c-Met) is a receptor tyrosine kinase that belongs to the MET (MNNG HOS transforming gene) family (Salgia, 2017). C-Met is encoded by the human *MET* gene, located on chromosome 7 (bands q21–q31) (Liu, 1998). As a kinase receptor, c-Met is a 190 kDa glycoprotein heterodimer localized on the surface of epithelial and endothelial cells, and it has two binding sites for its specific ligand, HGF/SF (hepatocyte growth factor or scatter factor). The first binding site contains the IPT3 and IPT4 domains, which have high affinity for the N domain of HGF. The second binding site is the SEMA domain, which has low affinity for the SPH domain of activated HGF (Stamos et al., 2004; Basilico et al., 2008). Binding of HGF can initiate stable c-Met homodimerization, which is mediated by adaptor proteins (GAB1 and GRB2), and then activates a number of key signaling pathways, including PI3K/Akt, Erk1/2, JAK/STAT, Src, Ras/MAPK, and Wnt/β-catenin (Imura et al., 2016; Pilotto et al., 2017), to induce cell proliferation, migration, invasion, and other biological effects (Ponzetto et al., 1994; Johnson and Lapadat, 2002) (Figure 1A). The HGF/c-Met axis is involved in biological and pathological processes such as embryogenesis, wound healing, and hepatic renal and epidermis regeneration (Parikh et al., 2014). The oncogenicity of c-Met is rarely caused by genetic alteration, and is more often due to upregulation of the wild-type gene (Trusolino et al., 2010; Gherardi et al., 2012). Amplification of c-Met was detected in patients with advanced solid cancers (Jardim et al., 2014). Furthermore, mutations, overexpression, or amplification of the *MET* gene in some tumor types resulted in aberrant HGF/c-Met axis activity, which induced cell motility and proliferation, promoted tumor development, and led to resistance to radiotherapy and targeted drug therapy in multiple cancers (Minuti et al., 2012; Barrow-Mcgee et al., 2016; Bahrami et al., 2017). Clinical trials of drug monotherapies targeted to c-Met have shown promising outcomes against multiple cancer types (Spigel et al., 2013; Solomon et al., 2014; Kogita et al., 2015). However, these medications cause significant side effects. Treatments that target the HGF/c-Met axis require further development.

Early studies have proved that besides proteins, there are lots of unique RNAs encoded by the genome are functional. Subsequent researches discovered a variety of non-coding RNAs (ncRNAs), including microRNAs (miRNAs) and long non-coding RNAs (lncRNAs) (Bejerano et al., 2004; Johnsson et al., 2014). Non-coding RNAs (ncRNAs) are involved in many signaling pathways associated with tumor aggressiveness and metastasis. MiRNAs are 20-22 nucleotide (nt) non-coding, highly conserved RNA molecules present in all human cells. MiRNAs can regulate 20–30% of all transcripts (Krol et al., 2010). The effect of specific miRNAs can be found in normal and cancer tissues, and in different cancer subtypes (Iorio and Croce, 2012). In the last 20 years, the role of miRNAs in oncogenesis has received increased attention. MiRNAs are encoded and transcribed as initial miRNA transcripts (pri-miRNAs), and processed to the precursor miRNAs (pre-miRNA) that harbor a stem-loop structure in nucleus. Then pre-miRNAs are processed into dsRNAs by RNase III enzyme DICER1, and merged into RNA-induced silencing complex (RISC) in cytoplasm. Only one strand of the dsRNA is preserved in RISC as miRNAs, while the other undergoes fast degradation (De Los Santos et al., 2019). MiRNAs can downregulate specific genes at the post-transcriptional level by interaction with the 3' UTR) of target messenger RNA (mRNA). They function in different ways via sequence complementarity: imperfect base pair match results in translational repression or mRNA cleavage, while perfect pairing leads to deadenylation (Ruvkun, 2001) (Figure 1B). Studies have suggested that miRNAs may be potential diagnostic and prognostic cancer biomarkers.

NcRNAs >200 nt in length are referred to as lncRNAs (Quinn and Chang, 2016). LncRNAs regulate various cellular processes including chromatin, transcriptional and post-transcriptional modification, and scaffolding. LncRNAs are ubiquitous in subnuclear domains and in the cytoplasm. MiRNAs regulate mRNA expression through degradation or silencing of mRNAs, while lncRNAs activate and repress genes via diverse mechanisms at the transcriptional or post-translational levels. MiRNAs and lncRNAs can interact to modulate the transcriptome (Yoon et al., 2014). Prevalent mechanism of action of lncRNAs in cancer is as miRNA decoys. LncRNAs can act as competitive endogenous RNAs (ceRNAs), decoys, or sponges that bind to specific miRNAs to prevent interactions with target mRNAs (Figure 1B). Some lncRNAs act as precursors of miRNAs to repress the downstream gene. Also, there are other lncRNAs can modulate mRNA through the complementary sequences (Salmena et al., 2011). Although lncRNAs are relatively stable, they are dysregulated in many diseases (Du et al., 2013). Increasing numbers of studies have focused on the functions of miRNAs and lncRNAs in homeostasis and maintenance of cell identity, pathogenesis, and carcinogenesis. Studies have shown that lncRNAs and miRNAs may act as independent master regulators of carcinogenesis, and interactions among lncRNAs, miRNAs, and specific mRNAs may be important in tumor-related processes.

A number of studies have correlated aberrant expression of lncRNAs, miRNAs, and c-Met with various cancers. In this review, we summarized the latest studies of the interplay between ncRNA (especially miRNAs and lncRNA) and the HGF/c-Met axis. In addition, we showed through this literature review that miRNAs and lncRNAs are critical regulators of c-Met in multiple cancers. Furthermore, the lncRNAs/miRNAs/c-Met axis may have potential as a target for treatment of cancer.

## The miRNAs/c-Met Axis in Cancer

Studies have shown that c-Met is a target gene of multiple miRNAs (Tables 1, 2) that play critical roles in controlling HGF/c-Met activity. This section summarizes recent studies of miRNAs that regulating c-Met.

**Table 1:** Anti-oncogenic miRNAs that target c-Met.

**miRs**	**Mechanisms (Inhibited pathway)**	**Functions**	**Disease or models**	**Reference**
miR-34a	c-Met/PI3K/Akt/mTOR	–	Triple-negative breast cancer	Hajalirezay Yazdi et al., 2018
	c-Met/PI3K/Akt	Inhibits cell growth and induced apoptosis; overcomes HGF-mediated gefitinib resistance	NSCLC (EGFR mutant)	Zhou et al., 2014
	c-Met	Extends recurrence-free survival of patients	Lung adenocarcinoma	Hong et al., 2015
	c-Met	Inhibits cell cycle progression, proliferation, and migration	Glioblastoma	Ofek et al., 2016
	c-Met and Notch	Inhibits cell proliferation, cell cycle progression, cell survival, and cell invasion	Glioblastoma	Li et al., 2009
	c-Met	Drives acute and late stages of radiation-induced fibrosis	Radiation-induced fibrosis (RIF)	Simone et al., 2014
	c-Met	Induces apoptosis and cell cycle arrest; suppresses tumor growth	Osteosarcoma	Zhao et al., 2016
	c-Met	Inhibits proliferation and metastasis	Osteosarcoma	Yan et al., 2012
	c-Met and AXL	Inhibits proliferation, invasion, and tumorigenicity; induces apoptosis	DMPM	El Bezawy et al., 2017
	c-Met	Suppresses cell growth, migration, and invasion; increases cellular apoptosis and caspase activity	HCC	Dang et al., 2013
	c-Met	Modulates proliferation and apoptosis *in vivo*; enhances cisplatin sensitivity *in vitro*	Gastric cancer	Zhang et al., 2016b
	c-Met	Attenuates proliferation, invasion, and metastasis	Gastric cancer	Wei et al., 2015
	c-Met/PI3K/Akt PDGFR	Inhibits growth, invasion, and metastasis	Gastric cancer	Peng et al., 2014
	c-Met	Inhibits proliferation and migration	Retinal pigment epithelial cells	Hou et al., 2013
	c-Met/Akt, Erk1/2	Suppresses proliferation and migration	Human lens epithelial cell	Feng et al., 2019
miR-34a miR-608	c-Met	Inhibits cell proliferation and invasion; induces apoptosis	Chordoma	Zhang et al., 2014
miR-34b	c-Met (MET1)	Decreases cell viability	Early Gastric Cancer	Yu, 2017
miR-34c	c-Met	Suppresses cell proliferation, migration, and invasion	NPC	Li et al., 2015
miR-206	c-Met/Akt/mTOR	Inhibits cell proliferation; induces cell apoptosis.	ESCC	Zhan et al., 2019; Zhang et al., 2019
	PAX3/c-Met/Akt/ Erk	Decreases cell proliferation and metastasis; increases apoptosis	Osteosarcoma	Zhan et al., 2019
	c-Met/Akt/mTOR	Inhibits proliferation and metastasis; induces apoptosis	Epithelial ovarian cancer	Dai et al., 2018
	c-Met	Delays cell-cycle progression, induces apoptosis, and impairs proliferation	HCC	Wu et al., 2017
	c-Met	Inhibits cell proliferation and migration; promotes apoptosis	HCC	Wang et al., 2019
	FMNL2 and c-Met	Suppresses proliferation; inhibits cell invasion and lung metastasis	CRC	Ren et al., 2016
	c-Met/PI3k/Akt/mTOR	EMT and angiogenesis	NSCLC	Chen et al., 2016b
	c-Met/PI3K/Akt/mTOR	Reverses cisplatin resistance	Lung adenocarcinoma	Chen et al., 2016a
	c-Met	Inhibits proliferation and migration	Gastric cancer	Zheng et al., 2015
	PAX3/c-Met	Reduces invasiveness and pulmonary metastasis	Gastric cancer	Zhang et al., 2015
	c-Met and EGFR	Inhibits cancer cell proliferation, migration, and invasion	NSCLC	Mataki et al., 2015
	c-Met	Activates apoptosis and inhibits tumor cell proliferation, migration, and colony formation	NSCLC	Sun et al., 2015a
	c-Met	Inhibits migration and invasion *in vitro*; inhibits proliferation and metastasis *in vivo*	Lung cancer	Chen et al., 2015b
miR-1 miR-206	c-Met and EGFR	Inhibits cell proliferation	HNSCC	Koshizuka et al., 2017
miR-1-3p miR-206	c-Met/Akt, Erk	Overcomes HGF-induced gefitinib resistance	Lung cancer	Jiao et al., 2018
miR-1	c-Met/Akt/mTOR	Reduces viability and inhibits proliferation	Prostate cancer	Gao et al., 2019
	c-Met	Inhibits proliferation and migration	Cervical cancer	Cheng et al., 2019
	c-Met	Inhibits cell proliferation and migration	Ovarian cancer	Qu et al., 2017
	c-Met	Suppresses cell growth	Osteosarcoma	Zhu and Wang, 2016
	c-Met/cyclin D1/CDK4	Suppresses proliferation; increases apoptosis	ESCC	Jiang et al., 2016b
	c-Met	Inhibits cell proliferation and migration	Gastric cancer	Han et al., 2015
	c-Met	Inhibits growth, replication potential, motility/migration, and clonogenic survival; induces apoptosis *in vitro*; inhibits tumor formation *in vivo*	Lung cancer	Nasser et al., 2008
miR-200a	HGF/c-Met	Suppresses migration and invasion; enhances radiosensitivity	NSCLC	Du et al., 2019
	EGFR and c-Met	Inhibits migration, invasion, and gefitinib resistance	NSCLC	Zhen et al., 2015
miR-365-3p	EHF/keratin 16 /β-integrin/c-Met	Decreases migration, invasion, metastasis, and chemoresistance	OSCC	Huang et al., 2019
miR-198	HGF/c-Met	Inhibits cell proliferation, migration, and invasion; induces apoptosis *in vitro*; overcomes resistance to radiotherapy; induces apoptosis *in vivo*	NSCLC	Zhu et al., 2018b
	HGF/c-Met	Inhibits migration and invasion	HCC	Tan et al., 2011
miR- 198 miR−206	c-Met	Reduces migration and invasion *in vitro*; reduces the number of lung metastases and prolongs overall survival *in vivo*	Osteosarcoma	Georges et al., 2018
miR-320	NRP-1/ HGF/c-Met	Inhibits cell proliferation; reduces cell migration *in vivo*; suppresses tumorigenesis, tumor growth, and lung metastasis *in vitro*	Cholangiocarcinoma	Zhu et al., 2018a
miR-203	DKK/c-Met	Reduces migration and invasion	Lung adenocarcinoma	Zhang et al., 2018
miR-410	c-Met	Inhibits proliferation and invasion	Glioma	Chen et al., 2012
miR-148-3p	c-Met	Suppresses invasive and proliferative capacity	Epithelial ovarian cancer	Wang W. et al., 2018
miR-485	c-Met/RAC/Akt	Suppresses proliferation and invasion	Cervical cancer	Wang S. et al., 2018
miR-133b	Sox9/c-Met	Suppresses clonogenic ability and metastatic traits *in vitro*; suppresses carcinogenesis and pulmonary metastasis *in vivo*	Breast cancer	Wang Q. Y. et al., 2018
miR-598	MAAC1/c-Met/Akt	Inhibits cell proliferation and invasion	Glioblastoma	Wang N. et al., 2018
miR-32-5p	TR4/HGF/c-Met /MMP2, MMP9	Suppresses metastasis	ccRCC	Wang M. et al., 2018
miR-140-5p	c-Met/Akt/mTOR	Inhibits cell growth	Retinoblastoma	Liao et al., 2018
miR-182	c-Met	Inhibits the EMT and metastasis	Lung cancer	Li et al., 2018b
	c-Met	Sensitizes TMZ-induced apoptosis; promotes cell differentiation; reduces proliferation	Glioblastoma	Kouri et al., 2015
miR-152	c-Met/PI3K/Akt	Represses cell proliferation, colony formation, migration, and invasion *in vivo*; suppresses tumor growth *in vivo*	OSCC	Li et al., 2018a
	c-Met/PI3K/Akt	Decreases cell growth; increases apoptosis	LMS and UPS	Pazzaglia et al., 2017
miR-27b	c-Met/PI3K/Akt	Suppresses cell viability and proliferation *in vitro*; inhibits tumor growth *in vivo*.	DLBCL	Jia et al., 2018
	c-Met	Inhibits proliferation, migration, and invasion	NSCLC	Zhou et al., 2017
miR-454	c-Met	Inhibits tumor growth and invasion	Osteosarcoma	Niu et al., 2015
miR-454-3p	c-Met	Inhibits invasion and migration	Cervical cancer	Guo Y. et al., 2018
miR-449a	c-Met/Ras/Raf/Erk	Inhibits cell growth	HCC	Cheng et al., 2018
miR-449b	c-Met	Inhibits proliferation	Thyroid carcinoma	Chen et al., 2015a
miR-449c	c-Met	Inhibits cell growth and promotes apoptosis	Gastric carcinoma	Wu Z. et al., 2015
miR-146a	c-Met	Reduces malignancy *in vitro*; prevents development of primary tumor and liver metastases	CRC	Bleau et al., 2018
miR-26a/b	HGF/c-Met	Inhibits metastasis	Gastric cancer	Zhang et al., 2017a
miR-23b	c-Met	Induces apoptosis	Cervical cancer	Yeung et al., 2017
	c-Met	Decreases severity of lesions	Cervical cancer	Campos-Viguri et al., 2015
miR-23b	c-Met	Inhibits cell migration and invasion	OSCC	Fukumoto et al., 2016
miR-27b	c-Met and EGFR	Inhibits cancer cell proliferation, migration, and invasion	Bladder cancer	Chiyomaru et al., 2015
miR-340	HGF/c-MET	Inhibits angiogenesis	MM	Umezu et al., 2017
	c-Met	Inhibits cell migration and invasion	Breast cancer	Wu et al., 2011
miR-181-5p	ETS1/c-Met	Improves prognosis	PDAC	Tomihara et al., 2017
	HGF/c-Met	Suppresses motility, invasion, and branching morphogenesis	HCC	Korhan et al., 2014
	c-Met	Suppresses motility, invasion, and branching morphogenesis	HCC	Korhan et al., 2014
miR-141	HGFR/c-Met	Inhibits proliferation and migration	CRC	Long et al., 2017
miR-489-3p	PAX3-c-Met	Inhibits metastasis	Osteosarcoma	Liu et al., 2017
miR-323-3p	c-Met/SMAD3/SNAIL	Inhibits EMT progression	Bladder cancer	Li et al., 2017a
miR-137	c-Met	Inhibits cell proliferation, colony formation, migration, and invasion; inhibits tumor progression	CRC	Chen et al., 2017b
	c-Met/Akt	Improves dexamethasone sensitivity	MM	Zhang et al., 2016a
miR-19a	c-Met	Reverses gefitinib resistance *in vitro* and *in vivo*; inhibits the EMT	NSCLC	Cao et al., 2017
miR-433	c-Met/CREB1-Akt/GSK-3β/Snail	Inhibits the EMT	Bladder cancer	Xu et al., 2016
miR-3666	c-Met	Decreases cell proliferation; increases cell apoptosis	Thyroid carcinoma	Wang et al., 2016
miR-329	c-Met	Inhibits cell proliferation, migration, and invasion; promotes apoptosis	Lung cancer	Sun et al., 2016
miR-130	c-Met	Not known	Prostate cancer	Nara et al., 2016
miR-16	HGF/c-Met	Inhibits proliferation and migration	Gastric cancer	Li et al., 2016b
	FGFR-1/MEK1/HGF	Reduces migration and tumor growth	Lung cancer	Andriani et al., 2018
miR-138	c-Met	Inhibits proliferation	Cervical cancer	Li et al., 2016a
miR-128	c-Met/PI3K/Akt	Reverses gefitinib resistance	Lung cancer	Jiang et al., 2016a
miR-31	c-Met/PI3K/Akt	Inhibits growth	Lung adenocarcinoma	Hou et al., 2016
miR-335-5p	NEAT1/miR-335-5p/c-Met	Inhibits proliferation and metastasis; promotes apoptosis	Pancreatic cancer	Cao et al., 2016
miR-335	c-Met	Inhibits migration	Breast cancer	Gao et al., 2015
miR-335 miR-1026	c-Met	–	NSCLC	Zhu et al., 2015
miR-122	c-Met	Suppresses cell proliferation and augments apoptosis; Prevents tumor cell colony formation and endothelial tube formation	HCC	Yang et al., 2015
miR-143	CD44 v3/HGF/c-Met	Inhibits migration and invasion	OSCC	Xu et al., 2015
miR-195	c-Met	Represses migration and invasion	Prostate cancer	Wu J. et al., 2015
miR-144	c-Met	Inhibits proliferation and invasion	Uveal Melanoma	Sun et al., 2015c
	c-Met	Inhibits metastasis	Gastric cancer	Liu J. et al., 2015
miR-144-3p	c-Met	Represses proliferation and invasion	Glioblastoma	Lan et al., 2015
miR-139-5p	c-Met	Inhibits proliferation and metastasis; promotes apoptosis	NSCLC	Sun et al., 2015b
miR-185	c-Met	Inhibits proliferation	Breast cancer	Fu et al., 2014
miR-409-3p	c-Met/Akt	Inhibits growth; induces apoptosis; reduces migration and invasion	Lung adenocarcinoma	Wan et al., 2014
miR-338-3p	MACC1/c-Met/Akt	Inhibits the EMT	Gastric cancer	Huang et al., 2015
miR-199a-3p	c-Met/Akt	Inhibits proliferation, adhesion, and invasiveness; suppresses peritoneal dissemination	Ovarian carcinoma	Kinose et al., 2015
	c-Met /Erk2	Inhibits cell proliferation and invasion; increases apoptosis	A549 cells (NSCLC)	Kim et al., 2008
miR-193a-5p	HGF/c-Met	Inhibits cancer aggressiveness	Ovarian cancer (Neo Adjuvant Chemotherapy)	Mariani et al., 2014
miR-7515	c-Met	Decreases proliferation and migration	Lung cancer	Lee et al., 2013
miR-101	c-Met	Suppresses motility	Bladder cancer	Hu et al., 2013

**Table 2:** Oncogenic miRNAs target c-Met.

**miRs**	**Mechanisms**	**Functions**	**Disease or models**	**Reference**
miR-29b-1-5p	synergizes with c-Met	Induces the EMT	OSCC	Kurihara-Shimomura et al., 2019
miR-222/221	c-Met/JNK/c-Jun/AP-1 Downregulates PTEN and TIMP3	Regulates TRAIL-resistance and enhances tumorigenicity	Lung cancer	Garofalo et al., 2009
miR-658	PAX3/c-Met	Induces metastasis	Gastric cancer	Wu et al., 2018
miR-K6-5p (KSHV)	CD82/c-Met	Expedites cell invasion and angiogenesis	Kaposi's sarcoma	Li et al., 2017b
miR-93	c-Met/PI3K/Akt	Stimulates cell proliferation, migration, and invasion; inhibits apoptosis	HCC	Ohta et al., 2015

### Antioncogenic miRNAs Target c-Met

#### miRNA-34 Family and c-Met

The miR-34 family, which includes miR-34a, miR-34b, and miR-34c, has been shown to inhibit cell cycle progression, epithelial-mesenchymal transition (EMT), metastasis, and stemness, and was shown to promote apoptosis, which resulted in suppression of tumor growth and carcinogenesis (Rokavec et al., 2014). All members of the miR-34 family negatively regulate c-Met (Figure 1D). Some studies have shown that miR-34 may be the primary miRNA regulator of c-Met. Mature miR-34, which lacks a 5'-phosphate, is inactive in cells. Phosphorylation of miR-34 resulting from DNA damage leads to activation of miR-34 (Salzman et al., 2016). MiR-34a and miR-34b levels are downregulated in non-small cell lung cancer (NSCLC) tissue compared to those in paired normal tissue. In addition, decreased levels of miR-34a have been associated with a higher risk of relapse, and lower levels of miR-34b expression have been associated with a higher frequency of lymph node metastasis (Gallardo et al., 2009). The miR-34 family inhibits the expression of c-Met, which is part of the p53 tumor suppressor network (Hwang et al., 2011). Wild p53 negatively regulates c-Met expression by transactivation of miR-34 or inhibition of SP1 binding to *MET* promoter (Hwang et al., 2011). On the other hand, HDM4, a potent inhibitor of p53, is repressed by miR-34 directly. Therefore, miRNA-34 and p53 form a positive feedback loop, resulting in inhibition of c-Met (Migliore and Giordano, 2009). Nuclear receptor subfamily 4 group A member 2 (NR4A2) is an orphan nuclear receptor that is upregulated in cancer and is involved in malignant biological properties. A study showed that overexpression of miR-34 or activation of p53 inhibited the expression of NR4A2. On the other hand, NR4A2 can suppress p53 and transcriptional targets, including miR-34, which suggested another feedback mechanism of p53/miR-34 (Hwang et al., 2011). The levels of miR-34a are significantly lower in hepatocellular carcinoma (HCC) than those in non-tumorous tissues, and are further reduced in HCC with metastasis or portal vein tumor embolus. Studies have shown that miR-34a suppressed cell growth, migration, and invasion, and increased cellular apoptosis and caspase activity by targeting c-Met in HCC cells (Dang et al., 2013). In addition, miR-34a downregulated Snail directly or through inhibition of HGF/c-Met, which promoted EMT in gastric cancer (Liu Y. W. et al., 2015). Furthermore, miR-34a attenuated proliferation and invasion in gastric cancer (Wei et al., 2015) and colorectal cancer (Luo et al., 2018). MiR-34a might also suppress brain tumors by targeting c-Met and Notch, which resulted in inhibition of cell proliferation, reduced cell survival, and decreased cell invasion of glioma and medulloblastoma cells, but not astrocytes (Li et al., 2009). A stable and efficient cationic carrier (dendritic polyglycerolamine) of mature miR-34a has been shown to inhibit cell cycle progression, proliferation, and migration of glioblastoma cells through modulation of c-Met (Ofek et al., 2016). Another study showed that overexpression of miR-34a suppressed tumor growth and metastasis of osteosarcoma (OS), possibly through downregulation of c-Met (Yan et al., 2012). Bioengineered miR-34a has showed to decrease the expression of c-Met and inhibited OS cell growth and invasion by inducing apoptosis and cell cycle arrest. This study confirmed the effectiveness and safety of bioengineered miR-34a using an orthotopic OS xenograft tumor mouse model (Zhao et al., 2016). Another study showed an inverse relationship between the expression of miR-34a-5p and c-Met in bone metastasis, but not in non-metastatic or metastatic breast carcinomas (Maroni et al., 2017). The expression level of miR-34a in triple-negative (MDA-MB-231) and HER2-overexpressing (SK-BR-3) cells was lower than that in normal breast cells (MCF-10A). MiR-34a has been shown to target c-Met and AXL in triple-negative breast cancer (Hajalirezay Yazdi et al., 2018). In addition, miR-34 has been increasingly studied because of its role in tumor growth, metastasis (Maroni et al., 2017), and drug resistance (Ghandadi and Sahebkar, 2016). Studies have also focused on miR-34a as a therapeutic agent and prognostic biomarker (Chen et al., 2017a).

#### miR-206 and c-Met

Recent evidence suggests that miR-206 inhibited HGF-induced EMT and angiogenesis in NSCLC via the c-Met/PI3k/Akt/mTOR pathway (Chen et al., 2016b). In other studies, miRNA-206, an inhibitor of c-Met and Bcl-2, was shown to promote apoptosis and inhibit tumor cell proliferation, migration, invasion, and colony formation in NSCLC (Sun et al., 2015a). MiRNA-206 has been shown to regulate cisplatin resistance and EMT in human lung adenocarcinoma cells by targeting c-Met (Chen et al., 2016a). In addition, miR-206 has been shown to sensitize HGF-induced gefitinib-resistant human lung cancer cells through inhibition of c-Met signaling and the EMT (Jiao et al., 2018). MiR-206 also has been shown to prevent the pathogenesis of hepatocellular carcinoma by modulating the expression of the *MET* proto-oncogene and cyclin-dependent kinase 6 in mice (Wu et al., 2017). Another study showed that miR-206 inhibited hepatocellular carcinoma cell proliferation and migration, but promoted apoptosis, by modulating the expression of c-Met (Wang et al., 2019). Furthermore, miR-206 expression was downregulated in GC cells, particularly in metastatic lesions, and loss of miR-206 promoted gastric cancer metastasis through activation of the PAX3/c-Met pathway (Zhang et al., 2015). Similarly, miR-206 was also significantly downregulated in CRC tissues, and a series of loss-of-function and gain-of-function assays were performed to demonstrate that miR-206 suppressed CRC cell proliferation and invasion by targeting FMNL2 and c-Met (Ren et al., 2016).

#### miR-1 and c-Met

MiR-1, which is abundant in cardiac and smooth muscles, is expressed in the lung and is downregulated in primary lung cancer tissues and cell lines. A previous study has reported that exogenous miR-1 significantly reduced the expression of c-Met, and enhanced sensitivity to doxorubicin-induced apoptosis (Nasser et al., 2008). The seed sequences of miR-1 and miR-206 are identical, and both inhibited c-Met and the downstream Akt and Erk pathways, and HGF-induced EMT, and increased gefitinib sensitivity in lung cancers (Koshizuka et al., 2017; Jiao et al., 2018). Downregulation of miR-1 and upregulation of c-Met have also been reported in gastric, prostate, cervical, and ovarian cancers (Han et al., 2015; Qu et al., 2017; Cheng et al., 2019; Gao et al., 2019). MiR-1 directly inhibits the expression of c-Met/Akt/mTOR and suppresses cell proliferation, migration, and EMT.

#### Other Antioncogenic miRNAs and c-Met

Most miRNAs in c-Met signaling are antioncogenic. Besides above, there are antioncogenic miRNAs include miR-410, -598, -182, and -144-3p in nervous system (Chen et al., 2012; Kouri et al., 2015; Lan et al., 2015; Wang N. et al., 2018); miR-140-5p in retinoblastoma (Liao et al., 2018) and miR-144 in uveal melanoma (Sun et al., 2015c); miR-365-3p, -152, -23b, -27b, and -143 in oral squamous cell carcinoma (OSCC) (Xu et al., 2015; Fukumoto et al., 2016; Li et al., 2018a; Huang et al., 2019). MiR-449b and -3666 are downregulated in thyroid cancer (Chen et al., 2015a; Wang et al., 2016); and miR-133b, -340, -335, and -185 are inhibit tumor progression in breast cancer (Wu et al., 2011; Fu et al., 2014; Gao et al., 2015; Wang Q. Y. et al., 2018). On account of the attention, a lot of miRNAs are found deficiency in lung cancer, including miR-200a, 168-198, -203, -182, -27b, -19a, -329, -16, -128, -31, -335, -1026, -139-5p, -105-3p338-3p, and miR-7515 in lung cancer (Kim et al., 2008; Lee et al., 2013; Wan et al., 2014; Sun et al., 2015b, 2016; Zhen et al., 2015; Zhu et al., 2015, 2018b; Hou et al., 2016; Jiang et al., 2016a; Cao et al., 2017; Pazzaglia et al., 2017; Zhou et al., 2017; Andriani et al., 2018; Li et al., 2018b; Zhang et al., 2018; Du et al., 2019). The digestive system cancer are also found absence of some miRNAs, including miR-449c, -26a/b, -16, and -144 in gastric cancer (Huang et al., 2015; Liu J. et al., 2015; Wu Z. et al., 2015; Li et al., 2016b; Zhang et al., 2017a); miR-198, -449a, -181-5p, and -122 in HCC (Tan et al., 2011; Korhan et al., 2014; Cheng et al., 2018); miR-320 in cholangiocarcinoma (Zhu et al., 2018a); miR-335-5p in pancreatic cancer (Cao et al., 2016); miR-146a, -141, and -137 in colorectal cancer (Chen et al., 2017b; Long et al., 2017; Bleau et al., 2018). While miR-32-5p, -23b, -27b, -323-3p and -433 are reported inhibit c-Met signaling in urinary cancer (Hu et al., 2013; Chiyomaru et al., 2015; Xu et al., 2016; Li et al., 2017a; Wang M. et al., 2018). Moreover, miR-148-3p, -119a-5p, -485, -454-3p, -23b, -138, -130 and -195 are relevant to the malignancy of reproductive system (Mariani et al., 2014; Campos-Viguri et al., 2015; Kinose et al., 2015; Wu J. et al., 2015; Li et al., 2016a; Nara et al., 2016; Yeung et al., 2017; Guo Y. et al., 2018; Wang S. et al., 2018; Wang W. et al., 2018); and miR-198, -206, -454, and -489-3p are relevant to the skeletal system tumor (Niu et al., 2015; Liu et al., 2017; Georges et al., 2018); miR-340, -137 and -27b in hematological malignancy (Zhang et al., 2016a; Umezu et al., 2017; Jia et al., 2018).

#### Oncogenic miRNAs and c-Met

Some miRNAs exert oncogenic effects through modulation of the HGF/c-Met axis (Figure 1F). Studies have shown that miR-29b-1-5p was upregulated in OSCC compared with normal oral epithelium, and this upregulation correlated with OSCC histological grade and poor prognosis. In addition, up-regulation of miR-29b-1-5p showed a synergistic effect with c-Met, resulting in induction of the EMT in OSCC cells (Kurihara-Shimomura et al., 2019). MiR-658 may have an opposite effects on the metastasis of gastric cancer (MGC) compared to that induced by miR-206. Serum levels of miR-658 in patients with distant MGC were significantly higher than those with no MGC, and overexpression of miR-658 has been shown to activate the PAX3/c-Met pathway to promote metastasis of GC cells (Wu et al., 2018). Interestingly, viral miRNAs may also be oncogenic. MiR-K6 is a Kaposi's sarcoma-associated herpes virus (KSHV)-encoded microRNA, and miR-K6-5p has been shown to specifically inhibit the expression of endogenous CD82, a metastasis suppressor, and directly interact with c-Met to inhibit its activation, resulting in endothelial cell invasion and angiogenesis (Li et al., 2017b). Tumor necrosis factor (TNF)-related apoptosis inducing ligand (TRAIL) is a member of the TNF-α family that induces apoptosis by binding to death receptors on the cell surface. Some scholars noted that NSCLC cells that overexpressed miR-221/222 were TRAIL-resistant and showed increased migration and invasion (Garofalo et al., 2008). They also found that c-Met conferred resistance to TRAIL-induced cell death and enhanced tumorigenicity of lung cancer cells by activation of miR-221 and miR-222 through the c-Jun transcription factor (Garofalo et al., 2009). In another study, low levels of miR-130a were detected in NSCLC, and miR-130a targeted c-Met and reduced TRAIL resistance through c-Jun-mediated down-regulation of miR-221 and miR-222 (Acunzo et al., 2012). Evidence is also proposed that c-Met activation increased miR-130b levels, inhibited androgen receptor expression, and promoted cancer spreading and resistance to hormone ablation therapy. They also showed that c-Met/miR-130b axis expression in exosomes isolated from peripheral blood was a non-invasive tool for active surveillance and therapy monitoring (Cannistraci et al., 2017).

## LncRNAs/c-Met Axis in Cancer

### Oncogenic lncRNAs Target c-Met

Most lncRNAs that target c-Met act as ceRNAs, decoys, or sponges, and bind to specific miRNAs to prevent suppression of c-Met (Salmena et al., 2011) (Table 3).

**Table 3:** Oncogenic lncRNAs target c-Met.

**lncRNAs**	**Target miRs**	**Mechanisms**	**Functions**	**Disease or models**	**Reference**
lnc-TALC	miR-20b-3p	c-Met	Induces O6-methylguanine-DNA methyltransferase, temozolomide (TMZ) resistance	Glioblastoma	Wu et al., 2019
lnc-MALAT1	miR-34a/c-5p, miR-449a/b	c-Met	Induces the proliferation and metastasis	Osteosarcoma	Sun Z. et al., 2019
lnc-XIST	miR-34a	c-Met /PI3K/Akt	Modulates cell proliferation and tumor growth	Thyroid cancer	Liu et al., 2018
lnc-GAPLINC	miR-34a/c	c-MET	Increases the migration, Invasion	Colorectal Cancer	Luo et al., 2018
lnc-TUG1	miR-144	c-Met	Promotes the transferring and invading capacity	Gastric carcinoma	Ji et al., 2016
lnc-H19	estrogen receptor	HGF/c-Met	Increases the resistances to Fulvestrant and Tamoxifen	ER+ breast cancer cells	Basak et al., 2018
	miR-675	c-Met	Enhances tumorigenesis and metastasis	Breast cancer	Vennin et al., 2015
	miR-675-5p	c-Met	Increases the proliferation, tumor development and progression	Glioblastoma multiforme	Angelucci et al., 2018
lnc-SNHG8	miR-152	c-Met	Promotes the proliferation	Endometrial carcinoma	Yang et al., 2018
lnc-NEAT1	miR-449b-5p	c-Met	Promotes the proliferation, invasion, and migration	Glioma	Zhen et al., 2016
	miR-335	c-Met	Suppresses sorafenib sensitivity	HCC	Chen and Xia, 2019
lnc-PVT1	miR-152	c-Met/PI3K/Akt	Enhances chemoresistance to gemcitabine	Osteosarcoma	Sun Z. Y. et al., 2019
FAM83H-AS1	–	c-Met/EGFR	Promotes the proliferation, migration and invasion	Lung cancer	Zhang et al., 2017b
lnc-HOTAIR	PRC2/miR-34a	HGF/c-Met /Snail	Promotes EMT	Gastric cancer	Liu Y. W. et al., 2015
lnc-KCNQ1OT1	miR-153	c-Met	Promotes cell proliferation and metastasis	Melanoma	Guo B. et al., 2018
lnc ARSR	miR-34/miR-449	c-Met	Promotes sunitinib resistance	RCC	Qu et al., 2016
lnc-Hand2	–	Nkx1-2/c-Met	Promotes liver repopulation	Hepatocytes	Wang Y. et al., 2018

#### LncRNA-H19 and c-Met

Lnc-H19, a precursor of miR-675, has been associated with carcinogenesis. Investigators reported that overexpression of lnc-H19/miR-675 enhanced the oncogenic action of breast cancer cells, and increased cell proliferation and migration *in vitro* and *in vivo*. A study showed that lnc-H19/miR-675 enhanced activation of c-Met and EGFR, which resulted in sustained activation of Akt and Erk, and increased proliferation and migration through direct binding to c-Cbl and Cbl-b mRNA (Vennin et al., 2015). In contrast, in endocrine therapy resistant (ETR) cells, lnc-H19 regulated ERα transcript and protein levels, and maintained levels of ERα following treatment with fulvestrant. Pharmacological inhibitors of Notch and HGF/c-Met signaling decreased lnc-H19 and ERα expression, which resulted in decreased resistance to Tam and fulvestrant in ETR cells (Basak et al., 2018), which may represent a novel therapeutic strategy for ETR. A recent study showed that lnc-H19/miR-675-5p and c-Met levels were elevated in cancer stem cells (CSCs) of glioblastoma multiforme compared to those in peritumoral tissue (Angelucci et al., 2018).

#### Lnc-X-Inactive Specific Transcript (XIST) and c-Met

A study showed elevated levels of XIST and reduced levels of miR-34a in thyroid cancer tissues and cell lines. XIST serves as an oncogenic lncRNA, and a ceRNA (sponging) for miR-34a as well. XIST may compete with c-Met for miR-34a binding, which promotes thyroid cancer cell proliferation and tumor growth (Liu et al., 2018).

#### Lnc-Nuclear Enriched Abundant Transcript 1 (NEAT1) and c-Met

NEAT1, a highly abundant lncRNA in the nucleus, was found to be present at higher levels in glioma than in non-cancerous brain tissues. NEAT1 acts as a molecular sponge for miR-449b-5p, which leads to upregulation of c-Met, resulting in glioma pathogenesis (Zhen et al., 2016). Recently, *in vivo* and *in vitro* studies have shown the potential treatments for sorafenib resistance in HCC (Chen and Xia, 2019). NEAT1 enhanced c-Met/Akt pathway signaling by negatively regulating miR-335, which resulted in sorafenib resistance.

#### Other Oncogenic lncRNAs and c-Met

Other oncogenic lncRNAs include lnc-TALC in glioblastoma (Wu et al., 2019); lnc-FAM83H in lung cancer (Zhang et al., 2017b); lnc-TUG1 and lnc-HOTAIR in gastric cancer (Liu Y. W. et al., 2015); lnc-Hand2 in liver cancer (Wang Y. et al., 2018); lnc-ARSR in renal cell cancer (Qu et al., 2016); lnc-GAPLINC in colorectal cancer (Luo et al., 2018); lnc-SNHG8 in endometrial cancer (Yang et al., 2018); lnc-MALAT1 and LNC-PV1 in osteosarcoma (Sun Z. Y. et al., 2019); and lnc-KCNQ1OT1 in melanoma (Guo B. et al., 2018).

### Antioncogenic lncRNAs That Target c-Met

In addition to most lncRNAs act as miRNAs sponges to upregulate the expression of c-Met, there are also some lncRNAs suppress the cancer progression by targeting c-Met through other pathways (Figure 1C). Reduced expression of XIST was observed in patients with breast cancer. Interestingly, XIST plays an antioncogenic role in breast cancer. Levels of XIST were inversely correlated with brain metastasis. Downregulation of XIST resulted in activation of c-Met and induction of the EMT, which resulted in increased secretion of exosomal miRNA-503 and suppression of T-cell proliferation, and promoted stemness of tumor cells (Xing et al., 2018). A previous study has attempted to explain that lncRNA-MIR22HG played a tumor suppressive role through dysregulation of the oncogenes *YBX1, MET*, and *p21*, which resulted in reduced cell survival and increased cell death signaling in human primary lung tumors (Su et al., 2018). Another study showed that loss of lncRNA-MEG3 was associated with increased expression of c-Met in pancreatic neuroendocrine tumors (PNETs). They also showed that MEG3 bound to unique genomic regions in and around the c-Met gene, which may be a potential therapeutic target to increase pancreatic islet beta-cell expansion to ameliorate beta-cell loss in diabetes (Iyer et al., 2017) (Table 4).

**Table 4:** Antioncogenic lncRNAs target c-Met.

**lncRNAs**	**Target miRs**	**Mechanisms**	**Functions**	**Disease or models**	**Reference**
lnc-XIST	miR-503	MSN/c-Met	Inhibits brain metastasis	Breast cancer	Xing et al., 2018
lnc-MIR22HG	–	c-Met	Blocks both cell survival and cell death	Lung cancer	Su et al., 2018
lnc-MEG3	–	HGF/c-Met	Enhances cell proliferation	Pancreatic neuroendocrine tumor	Iyer et al., 2017

### LncRNAs/miRNAs/c-Met Pathway

#### Oncogenic lncRNAs/miR-34a/c-Met Pathway

As mentioned above, miRNA-34 is crucial in HGF/c-Met signaling. Many studies have focused on lncRNAs, including lnc-XIST, lnc-MALAT1, lnc-GAPLINC, lnc-HOTAIR, and lnc-ARSR, as ceRNAs targeting to miR-34 (Figure 1D). Recently, scholars explored up-regulation of lnc-MALAT1 (lung adenocarcinoma transcript 1) in osteosarcoma tissues and cell lines, which was significantly associated with poor prognosis (Sun Z. et al., 2019). The results of this study suggested that lnc-MALAT1 increased osteosarcoma proliferation and metastasis by competitively binding to miR-34a/c-5p and miR-449a/b. Increased expression of lnc-GAPLINC was detected in CRC compared to that in non-cancerous tissues, and identified a GAPLINC/miR-34a/c-Met axis involved in cell migration and invasion in CRC tissues and cells (Luo et al., 2018). Another study showed that lncRNA-HOX antisense intergenic RNA (HOTAIR) was highly expressed in gastric cancer, particularly in the diffuse type. This study showed that HOTAIR epigenetically repressed miR-34a by binding to PRC2, and then activated the HGF/C-Met/Snail pathway, which resulted in EMT and accelerated GC tumor metastasis (Liu Y. W. et al., 2015). It has been demonstrated that lncRNA activated in RCC with sunitinib resistance (lnc-ARSR) correlated with poor sunitinib response (Qu et al., 2016). Lnc-ARSR might modulate AXL and c-Met by competitively binding to miR-34/miR-449, which may be a potential therapeutic target for mitigation of sunitinib resistance.

#### LncRNAs/miRNAs/c-Met in Drug Resistance

In this section, resistance to current anticancer strategies used for treatment of lung, kidney, and pancreatic cancer is discussed (Figure 1E). Lnc-ARSR causes sunitinib resistance in RCC, lnc-H19 promotes resistance to fulvestrant and tamoxifen in breast cancer, and lnc-NEAT1 enhances resistance to sorafenib in HCC. Other lncRNAs may promote drug resistance, and may be predictors and potential targets for cancer treatment. It is interesting to observe that lnc-TALC (temozolomide-associated lncRNA in glioblastoma recurrence) was required for temozolomide (TMZ) resistance and glioblastoma recurrence. Lnc-TALC, which was regulated by the Akt/FOXO3 axis, elevated c-Met expression by competitively binding to miR-20b-3p (Wu et al., 2019). Similarly, lncRNA-PVT1 (Plasmacytoma Variant Translocation 1) played a vital role in chemoresistance of osteosarcoma cells. PVT1 has been shown to target and downregulate miR-152, which results in c-Met/PI3K/AKT pathway activation, and contributes to resistance to gemcitabine (Sun Z. Y. et al., 2019).

## miRNA, lncRNA-Based Therapeutics in c-Met-Related Cancer

**Figure 2:**
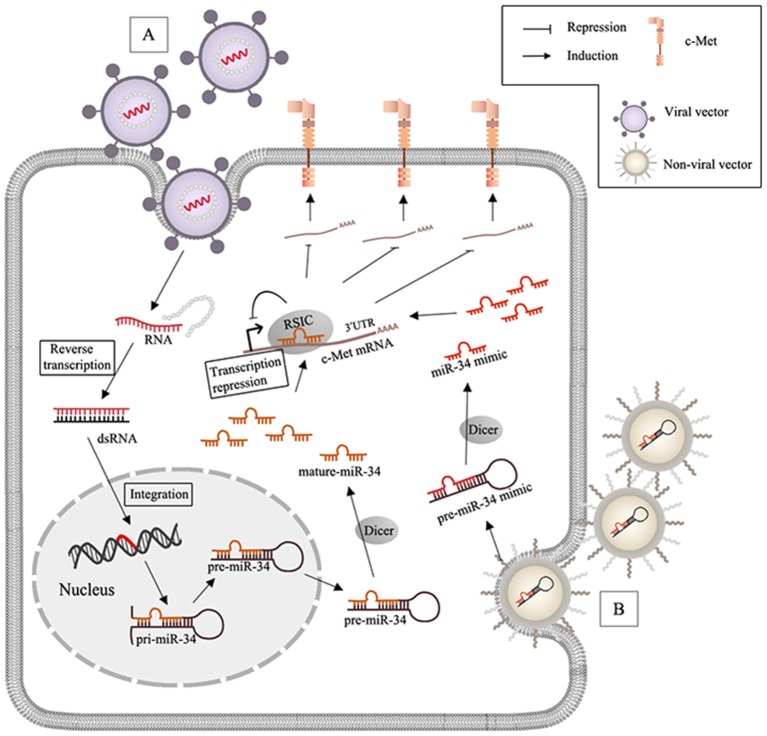
MiR-34 based therapy and delivery systems in c-Met-regulated cancers. **(A)** After virus (lentivirus or adenovirus) infects the target cells, RNA packaged by viral vectors is liberated with the wrapped protein and undergoes the reverse transcription to synthesize double strands RNA (dsRNA), and the genome of the host cell can integrate with dsRNA and generate miR-34; **(B)** non-viral nanoparticles including lipid- and polymeric carriers release the encapsulated miR-34 mimics after transfection.

Events such as c-Met gene mutation, overexpression, and amplification may be closely related to aberrant activation of HGF/c-Met signaling in human cancers (Barrow-Mcgee et al., 2016; Bahrami et al., 2017). Increasing preclinical evidence has allowed for development of specific molecular inhibitors, including anti-c-Met monoclonal antibodies and small molecule tyrosine kinase inhibitors (TKIs). Anti-c-Met monoclonal antibodies block binding to HGF, which prevents dimerization and induces degradation. Small molecules TKIs inhibit catalytic activity. Besides blocking monoclonal antibodies against c-Met or against the HGF ligand, c-Met inhibitors can be classified into two groups of TKIs: multi-kinase inhibitors and selective c-Met inhibitors. A number of clinical trials have been performed to evaluate drugs that target the HGF/c-Met axis, but the results of these trials have been unsatisfactory (Hughes and Siemann, 2018). The poor results of these studies suggested that HGF/c-Met signaling may be more complex than originally thought. Upstream regulation and additional functions of c-Met in progression of metastasis require further investigation. Many studies have evaluated the role of c-Met in cancer, but relationships among lncRNAs, miRNAs, and c-Met have not been characterized. Abundant clinical and ChIP analysis data has been generated to evaluate lncRNAs/miRNAs/c-Met, but few *in vivo* studies have been performed. Preclinical models and successful clinical trials are of critical importance. MiR-34a has been extensively studied and is a promising miRNA therapeutic candidate (Figure 2). Like miR-34, most miRNAs involved in HGF/c-Met signaling are anti-oncogenic, and the therapeutic approach via miRNAs mimics is considered as the main miRNA-based therapy. One of the biggest challenges is to ensure the delivery efficiency of miRNAs mimics to target cells. While RNase is abundant in serum and cytoplasm, miRNAs is short-lived in plasma (Sun et al., 2017). Moreover, chemically modify nucleotides can increase miRNAs expression and stability, but they are limited due to the low membrane penetrability. Hence, the main strategies are based on the nano-scaled carriers, which are divided to virus and non-virus. Viral vectors, including lentivirus, retrovirus and adenovirus are commonly used. The encapsulated genes can be transferred to the genome of target cells after virus infection. For instance, lentiviral miR-34a delivery system induced apoptosis in mammalian (MM) cells, reduced tumor size in MM xenografts (Di Martino et al., 2012), and prolonged prostate cancer animal survival (Liu et al., 2011). Among non-viral nanocarriers, lipid-based vector are frequently applied because of the high transfection efficiency, but they are limited on account of the poor stability in serum and the toxicity (Xue et al., 2015). To solve this, stable nucleic acid lipid particles (SNALPs) was synthesized, and SNALPs encapsulating miR-34a showed inhibition of MM xenograft growth with low toxicity (Di Martino et al., 2014). Besides, polymeric vector, another non-viral delivery system with the low immunogenicity and cytotoxicity is also used to inhibit the tumor via integrating miR-34a (Lin et al., 2019). The first miRNA-associated therapeutic drug, MRX34, was a lipid nanoparticle loaded with miR-34 mimics, and was tested in clinical trials (NCT01829971 and NCT02862145) (Beg et al., 2017). However, MRX34 was quickly withdrawn because of immune-related adverse events. Therefore, it is important to evaluate the safety of nanoparticles. Other non-viral nano particles are also used as delivery systems for miRNA therapeutics, like EnGeneIC Delivery Vehicle (EDV) nanocells (Taylor et al., 2015), synthetic polyethylenimine (PEI) (Akhtar and Benter, 2007), dendrimers (Duncan and Izzo, 2005), cyclodextrin (Gonzalez et al., 1999), chitosan (Ragelle et al., 2013), N-acetyl-D-galactosamine (Nair et al., 2014). Furthermore, it is important to develop reliable and effective approaches to development of lncRNA-based therapeutics. As discussed above, lots of known lncRNAs in the c-Met signaling are oncogenic. In order to silence oncogenic lncRNAs, three approaches might be used to promote their selective degradation of the lncRNAs, including: RNA interference (RNAi), Rnase H active antisense oligonucleotides (ASOs) and Clustered Regularly Interspaced Short Palindromic Repeats/CRISPR-Associated Protein 9 (CRISPR/Cas9) system; RNAi utilizes the RISC containing a specific siRNA to degrade the targeted lncRNA in cytoplasm (Dorsett and Tuschl, 2004). Rnase H ASOs use endogenous RNase H1, which cleaves the RNA in an RNA/DNA heteroduplex, to reduce the accumulation of lncRNA transcripts or block the functional domains (Ulitsky et al., 2011; Kurian et al., 2015) and CRISPR/Cas9 platform is a powerful genome-editing tool, which is already successfully applied to facilitate targeted genetic mutations. The endonuclease Cas9 is recruited to specific genomic sites via a sequence-dependent CRISPR RNA (crRNA) and a sequence-independent trans-activating CRISPRRNA (tracrRNA) (Jinek et al., 2012; Doudna and Charpentier, 2014). LncRNA-targeting efficiency and knockout confirmation have been done *in vivo*, which is a promising therapeutic technology (Hansmeier et al., 2019). Advances in delivery strategies may allow for development of novel lncRNA- and miRNA-based therapies, and the lncRNAs/miRNAs/c-Met axis may contain biomarkers for early detection and clinical management of patients with different types of cancers.

## Conclusions and Perspectives

Studies have shown that c-Met is associated with tumor formation, progression, and metastasis. Recent studies have highlighted the HGF/c-Met axis as a promising therapeutic target for treatment of metastatic tumors. Metastatic dissemination represents a critical problem in the treatment of malignancy, and results in poor prognosis and increased mortality in patients with cancer. The metastatic process includes growth and proliferation of the primary tumor, and migration, adhesion, invasion, and survival at distal sites. A number of lncRNAs and miRNAs that target c-Met may play different roles in carcinogenesis through various molecular mechanisms, including up-regulation or mutation of the *MET* gene. The relationships among lncRNAs, miRNAs, and c-Met in cancer require further characterization. Studies that evaluate the effect of specific lncRNAs or miRNAs on c-Met in different types of cancer are required to determine the therapeutic potential of targeting this signaling pathway.

Studies have shown that ncRNAs/HGF/c-Met signaling is involved in many physiological and pathological processes, and most miRNAs are anti-oncogenic and most lncRNAs are oncogenic. LncRNAs act as ceRNA, decoys, or sponges and bind to specific miRNAs, to prevent interactions with c-Met, which results in malignancy. Studies have shown that c-Met may drive oncogenesis and promote resistance to targeted therapies such as EGFR, VEGFR, HER2, and BRAF inhibitors (Engelman et al., 2007; Shojaei et al., 2010; Chapman et al., 2011; Saito et al., 2015). We reviewed the role of the lncRNA/miRNA/c-Met axis in drug resistance in specific cancers. Translation of molecular mechanisms of cancer resistance to clinical practice is necessary to develop therapeutic strategies to prevent or overcome resistance to targeted therapies, and to identify patient populations who are more likely to benefit from treatment with ncRNAs/HGF/c-Met targeted therapies.

**Figure 3:**
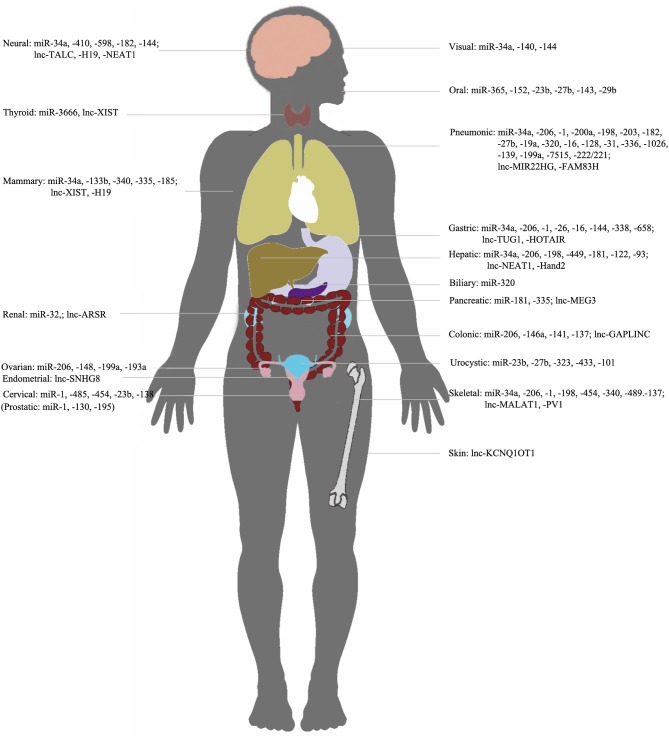
NcRNAs/c-met in different organs. Representation of organ-specific lncRNAs or miRNAs in c-Met regulation in cancer.

In conclusion, overexpression of c-Met can be induced by upstream lncRNAs and/or miRNAs in various types of tumors (Figure 3). Clinical trials of ncRNAs/c-Met for mitigation of specific drug resistance have shown promising results. In this review, we discussed interactions among lncRNAs, miRNAs, and c-Met, and the relationships of these interactions with tumorigenesis and progression of various cancers. We also reviewed molecular mechanisms associated with the onset and development of cancer, and discussed novel diagnostic therapeutic approaches. Further identification of tissue-specific lncRNAs and miRNAs that directly or indirectly regulate c-Met, and characterization of the associated molecular mechanisms, are necessary.

## Author Contributions

HZ and ST drafted the manuscript. FZ revised the manuscript. AS and JL reviewed and modified the manuscript. All authors agreed on the final version.

### Conflict of Interest

The authors declare that the research was conducted in the absence of any commercial or financial relationships that could be construed as a potential conflict of interest. The handling editor and the authors are affiliated with Zhejiang University, Hangzhou, but do not have any collaborations.
